# Self-exclusion from gambling: A toothless tiger?

**DOI:** 10.3389/fpsyt.2022.992309

**Published:** 2022-09-23

**Authors:** Ludwig Kraus, Johanna K. Loy, Andreas M. Bickl, Larissa Schwarzkopf, Rachel A. Volberg, Sara Rolando, Veera E. Kankainen, Matilda Hellman, Ingeborg Rossow, Robin Room, Thomas Norman, Jenny Cisneros Örnberg

**Affiliations:** ^1^Department of Public Health Sciences, Centre for Social Research on Alcohol and Drugs, Stockholm University, Stockholm, Sweden; ^2^IFT Institut für Therapieforschung, München, Germany; ^3^Institute of Psychology, ELTE Eötvös Loránd University, Budapest, Hungary; ^4^School of Public Health and Health Sciences, University of Massachusetts Amherst, Amherst, MA, United States; ^5^Eclectica, Institute for Research and Training, Torino, Italy; ^6^University of Helsinki Centre for Research on Addiction, Control and Governance (CEACG), University of Helsinki, Helsinki, Finland; ^7^Norwegian Institute of Public Health, Oslo, Norway; ^8^Centre for Alcohol Policy Research, La Trobe University, Melbourne, VIC, Australia; ^9^Australian Research Centre in Sex, Health and Society, La Trobe University, Melbourne, VIC, Australia

**Keywords:** self-exclusion, gambling, harm, responsible gambling, public health, legal regulations

## Abstract

While there is evidence for self-exclusion (SE) as an individual-level harm reduction intervention, its effects on reducing harm from gambling at the population level remain unclear. Based on a review of national legal frameworks and SE programs, including their utilization and enforcement in selected high-income societies, the present analysis aims to explore the reach and strengths of SE in the protection of gamblers in these jurisdictions. It places particular emphasis on SE programs' potential to prevent and minimize gambling harm at the population level. The overview examined SE in Finland, Germany, Italy, Massachusetts (USA), Norway, Sweden, and Victoria (Australia). These jurisdictions differ considerably in how gambling is regulated as well as in how SE is implemented and enforced. The reach and extent of enforcement of SE apparently vary with the polity's general policy balance between reducing gambling problems and increasing gambling revenue. But in any case, though SE may benefit individual gamblers and those around them, it does not appear to be capable of significantly reducing gambling harm at the population level. To render SE programs an effective measure that prevents gamblers and those linked to them from financial, social, and psychological harm, utilization needs to be substantially increased by reforming legal regulations and exclusion conditions.

## Introduction

Land-based gambling and more recently online gambling have increased in many parts of the world partly as the result of increasing liberalization and deregulation of gambling. Especially online gambling, previously illegal in most countries, has been legalized within existing schemes or by issuing licenses to national providers, resulting in an only partly regulated online market (1). The Gross Gambling Revenue, defined as the sum of all money gambled minus the wins returned to gamblers, is estimated to have almost doubled in Europe between 2003 and 2018, with an increase from 56 to over 100 billion EUR (2). In parallel, the revenues gained by states *via* a monopolistic position or by taxing licensed gambling provide reliable resources to fund welfare programs and other public expenses (3). For instance, about 60% of support for the cultural sector and 80% of support for sports activities in Finland stem from gambling-generated revenues (2). In Australia, revenue from gambling taxes is estimated to account for 8.4% of the Victorian state tax revenue (4). However, along with the substantial growth of the gambling market, concerns about harms from gambling emerge, along with calls for measures to keep a balance between the societal benefits, the spread of fraud and crime, and the harms associated with gambling (5–7).

Several measures that may help in reducing harm to the gambler have been discussed within the framework of “responsible gambling” (RG). This conceptualization is used by the industry to handle the two sides of the coin: gambling as a fun activity and the risk of harm from gambling (8, 9). RG measures put forward to limit harm from gambling include, amongst others, protective behavioral strategies such as self-exclusion, time and monetary limit setting, or card-based gambling programs, that allow individuals to load a predetermined amount of money onto a card (10). In contrast, the public health approach aims at reducing supply, for instance by removing gambling machines or reducing operating hours (2, 11). Although there is sound evidence for the impact of supply reduction measures on the prevalence of gambling and gambling harm (12–14), the revenue of the commercial gambling industry, as well as the maintenance of taxation revenue by governments, are two strong interests that are diametrically opposed to the goal of reducing harm by reducing supply (15). As supply reduction measures jeopardize the expansion of the gambling industry and reduce governmental funds for public services, individual-level measures targeting the gambler have become more politically popular. From the perspective of the gambling industry, such measures have the added benefit of diverting policy attention from the industry's promotion and incentives by pointing to deficiencies in the individual gambler. One set of such measures is the widely adopted self-exclusion (SE) measure, which offers gamblers a choice to ban themselves from particular gambling venues or from land-based as well as online gambling. SE from gambling is primarily an individualized harm reduction measure that aims at preventing gamblers from further financial, social, and psychological distress (16, 17).

Commercial casinos and gambling companies frequently offer SE, permitting individuals to ban themselves from entering specific venues or using specific services. More recently, several SE programs have evolved toward an individual assistance model where enrolees are offered not only debt counseling but also psychological support and addiction treatment (18–21).

By SE programs and provisions, we are here referring to regulations or a gambling provider's rule that allows a gambler to request not being permitted to engage in gambling with a specified provider or category of providers. Usually, a written and signed application is required, and the provider undertakes to refuse any attempt by the self-excluder to gamble with the provider for the period specified in the application. Thus, the provider's staff is expected to refuse entry to a gambling site or platform if the self-excluder is identified in or while seeking entry to the site or platform. In some jurisdictions, the SE program is legislated or otherwise officially sanctioned with penalties for non-compliance by a gambling provider. But while there is growing evidence of SE programs having the potential to be an effective individual-level harm reduction intervention (10, 22–24), their effects on reducing harm from gambling at the population level are questionable (25).

Considering the substantial differences in how jurisdictions regulate gambling and implement control measures, the present study aims to analyze and compare the approach, implementation, and scope of legal frameworks and SE programs in a purposive selection of high-income countries or states. Secondly, we pay special attention to SE in the framework of “responsible gambling” to address gambling harm: whether and under what conditions SE may have an effect on problem gambling in the population as a whole. The countries/states that were chosen for this overview are Finland, Germany, Italy, Massachusetts (USA), Norway, Sweden, and Victoria (Australia), representing a broad spectrum of regulatory policies and implementation of SE regulations. In each of the seven jurisdictions available information on legal frameworks of gambling and SE regulations, including registers, length and termination, utilization and enforcement was collected by a team which is a partner of the project “Responding to and Reducing Gambling Problems Studies (REGAPS).”

## Legal frameworks

The information on frameworks for gambling regulation is summarized by country/state in Table 1. Gambling in the legal context is defined as placing something of value at risk in the hopes of gaining something of greater value (26). While Finland and Norway maintain a state monopoly (or another strongly regulated monopoly) on all or some forms of gambling, in Italy, Massachusetts, and Victoria gambling is fully or partially licensed to commercial providers. In Germany and Sweden, a state monopoly and a licensing system exist in parallel.

**Table 1:** Summary of gambling frameworks by country.

**Country/State**	**Gambling regulation system**	**Gambling laws/acts**	**Gambling authority**
Finland	Monopoly (Government-owned agency, Veikkaus)	The lotteries act^1^	Ministry of Internal Affairs
Germany	Monopoly excluding commercial amusement machines with prizes (AWP). The gambling public monopoly consists of (a) casinos (Spielbanken), (b) the German lottery association, (c) TV lottery as well as (d) class lottery. AWPs, technically a specific type of slot machine do not fall under the state's monopoly but are subject to the German Industrial Code^2^.	State treaty on gambling (STG) for the State gambling monopoly ^3^: the fifth amendment of the German gambling ordinance (AGO) for AWP^4^	Various agencies on federal state level (16 federal states)
Italy	Concessions. The operation of gambling is however considered a state monopoly in the sense that the state transfers the operation to private companies.	Fragmented legislative framework (no framework law) mostly corollaries of financial and stability laws	Agenzia delle Dogane e Monopoli (ADM), controlled by the Ministry of Economy and Finance
Massachusetts (USA)	Licenses for casinos and racing; state monopoly for lottery. Online gambling is illegal at the time of this writing.	Expanded gaming act 2011	Massachusetts Gaming Commission (MGC) (casinos, racing). Massachusetts State Lottery Commission (lottery)
Norway	Monopoly. With the exceptions of private lotteries and bingos, only Norsk Tipping and Norsk Rikstoto are by law allowed to provide gambling. Casino, roulette, poker (with some exceptions), pyramid games, and chain game are forbidden in Norway.	The lottery act^5^, The gaming scheme act^6^ and The Totalizator Act^7^	The Norwegian gaming authority (Lotteritilsynet)
Sweden	Licenses. The six different types of licenses contained in the Gambling Law are (1) State gambling (land-based Casino, EGMs, some lotteries), (2) Gambling for public benefit purposes (some lotteries, land-based bingo), (3) Online gambling, (4) Betting, (5) Land-based commercial gambling, and (6) Gambling on ships in international traffic. Forms of gambling that are exposed to competition are online gambling, betting, land-based commercial games such as restaurant casinos and card games in tournament form, as well as slot machines and casino games on ships in international traffic.	Swedish Gambling Act (2018:1138); The Swedish Gambling Ordinance (2018:1475)	The Swedish Gambling Authority (Spelinspektionen)
Victoria (Australia)	Licenses. The casino is a private monopoly licensed by the state. Other legal gambling provision is also licensed by the independent state gambling and casino control commission.	Gambling regulation act^8^	Victorian gambling and Casino control commission (VGCCC) as of January, 2022^9^

1 https://www.Finlex.fi/fi/Laki/Kaannokset/2001/En20011047;

2 Ludwig et al. 2012 (44);

3 State treaty on the re-regulation of gaming in Germany (State Treaty on Gaming 2021—GlüStV 2021: https://ec.Europa.eu/Growth/Toolsdatabases/Tris/de/Search/?Trisaction=Search.Detail&Year=2020&num=304;

4 The fifth amendment of the German gambling ordinance (AGO): https://www.Gesetze-Im-Internet.de/Spielv/Index.Html;

5 The lottery act (Lov om Pengespill av 28.02.1992, nr 103) regulates private lotteries in various forms, plus the lottery game extra, provided by Norsk Tipping: https://Lovdata.no/Dokument/NL/lov/1995-02-24-11;

6 The gaming scheme act which regulates all games that are currently provided by Norsk Tipping (Except Extra): https://Lovdata.no/Dokument/NL/lov/1992-08-28-103;

7 The Totalizator act (Horse Race Bets) (Lov om Veddemål ved Totalisator av 07.01.1927, nr 3 Regulates all games currently provided by Norsk Rikstoto: https://Lovdata.no/Dokument/NL/lov/1927-07-01-3;

8 Gambling regulation act, Australia Victoria, March 29, 2021: https://Content.Legislation.vic.gov.au/Sites/Default/Files/2021-03/03-114aa088%20authorised.pdf;

9 https://www.Legislation.vic.gov.au/in-Force/Acts/Victorian-Commission-Gambling-and-Liquor-Regulation-Act-2011/011; https://www.Govtmonitor.com/Page.php?Type=Document&id=3372020.

State monopolies, however, are not without exceptions. In Norway, for instance, private lotteries and bingos provide a particular form of gambling in addition to two monopoly providers (Norsk Tipping and Norsk Rikstoto). The German gambling monopoly includes casinos, lotteries, sports betting, and electronic gambling machines (EGMs) but excludes commercial amusement machines with prizes (AWP), which are offered by private enterprises. With the 4^th^ revision of the German State Treaty on Gambling that came into force in July 2021, online gambling, including online sports betting, has been widely legalized and opened for commercial providers within a licensing system. Similarly, online gambling in Sweden is also subject to licensing. The new license system went into force on January 1, 2019 in the context of the Swedish Gambling Act of 2018 giving Sweden a new re-regulated gambling market. The non-competitive forms are monopolized by the state or licensed to non-profit “good cause” organizations. Forms of gambling that are subject to competition are online gambling and land-based games such as card games.

In Italy, the gambling market is based on concessions, a type of license that allows the holder to act as a proxy for the state while exonerating it from responsibility for negative externalities caused by the activity (27). This includes all types of games as well as EGMs that are also placed in general venues such as tobacco shops and bars. Concessions are granted through public tenders. In Victoria and Massachusetts gambling is formally license-based. In Victoria, casino games are offered within the scope of a private monopoly that is issued by the state to Crown Melbourne. EGMs are licensed not only in the casino but also in taverns (“hotels”) and sports and community clubs. In Massachusetts, licenses apply to casinos, racing, bingo, and charitable events, while the state maintains its monopoly on lotteries.

The main differences in gambling regulations in these countries/states consist in the scope of monopolization of gambling, the extent of the market that is shared with or entrusted to private providers *via* licenses or concessions, the modalities of online gambling provision, and the share of the online market that is not monopolized or licensed. In the late 1990s, in the context of the growing internet gambling market, the European Union (EU) repeatedly questioned member states' monopolies and their compatibility with European Community law (28). The private gambling industry's demand for deregulation and access to markets regulated by national monopolies was strengthened by the argument for free movement of goods and services in the European internal market and similar global trends (29). As member states had justified their gambling monopolies by their ability to provide revenues for the public good in the form of charities, grants, or taxes, and by preventing fraud, money laundering, and black-market gambling, the European Commission argued that using gambling revenue for the common good cannot be the reason for a monopoly. It furthermore required proof from the monopoly providers that the stated objective to prevent gambling problems was genuine (28).

Subsequently, member states emphasized the prevention of societal and individual problems as an important justification for maintaining the gambling monopoly but had to find means to allow access to internet gambling providers. Countries had to develop strategies suited to supporting the line of argumentation that a (partially) regulated online gambling market could curb the previous black market and steer online gambling into controllable channels. Consequently, in Finland, Norway, and Sweden (until 2019), the increased focus on gambling-related problems and emphasis on the responsible nature of monopoly-based systems to tackle these problems made it possible to keep the monopoly and even expand its activities to the Internet (1, 30). Recently, Germany opened the sports betting market for commercial providers and now accepts online provisions of sports and horse betting, casino games, virtual gambling machines and poker (31). Other countries like Italy adapted their regulations for foreign online operators to apply for gambling licenses. Operators do not have to be government-owned or conduct their business through a company registered in Italy (27). Online gambling in Victoria is licensed while in Massachusetts it is illegal.

It remains unclear how effectively the different regulatory regimes contribute to reducing gambling-related harm. According to a recent literature review, monopolistic regimes apparently perform somewhat better than license-based regimes in preventing problem gambling and limiting gambling in general (32). Because of the significant differences across the included monopolistic systems, the authors argued that other factors such as “availability, accessibility, scope of preventive work, responsible gambling policies, the existence of a sufficiently resourced independent monitoring body, as well as the implementation of a public health approach to gambling may better predict the levels of harm in society” than a monopoly (32) (p.232). It also tends to matter where the monopoly is located in the governmental structure—for instance, in the jurisdiction of the finance department or of a health or welfare department. These findings are in accordance with historical experience with state monopolies of markets for other attractive but problematic commodities—such as psychoactive substances (33).

## Self-exclusion regulations

An overview of SE regulations including provision and implementation, the existence of a central register, the individual choices of temporary and permanent bans, and the scope of utilization and control for the seven jurisdictions is provided in Table 2. In all jurisdictions, customers can self-exclude from online or land-based casino games and EGMs or both. Lotteries are generally excluded from SE provision. The reach of SE by interested gamblers differs between jurisdictions by type of game and whether they are offered online or land-based or both. However, while land-based provision of gambling is regulated in all jurisdictions by a state monopoly or a license/concession system, SE for online gambling only applies to providers that hold a license in the respective jurisdiction. Hence, the—generally unrecorded—fraction of total online gambling that is unregulated (unlicensed online games offered from abroad) needs to be considered. For example, in Norway, unrecorded provision is estimated at about one-third of all gambling (34).

**Table 2:** Summary of voluntary self-exclusion regulations (outreach/provision, implementation, central register) by country/state.

**Country/State**	**Outreach/Provision**	**Implementation**	**Self-exclusion register**
Finland	Land-based casinos: self-exclusion with an entry ban. Online casino: self-exclusion from certain or all of Veikkaus' online games. Gambling locations^1^: self-exclusion from EMGs is possible. Veikkaus' gambling arcades^2^: self-exclusion from EMGs is possible. The customer may agree with an individual gambling arcade on an entry ban to that arcade.	*Land-based casinos*: A self-ban on entry will be agreed with the casino staff. The casino staff will block entry if the customer has a valid self-ban. *Online casino:* The customer can set a self-ban electronically or ask Veikkaus' customer service to set a ban. Self-exclusion is monitored electronically. *Veikkaus*' *gambling arcades*: A self-ban on entry will be agreed with the arcade staff. Before 2021, the compliance with the agreements is not systematically monitored but changes in the Lotteries Act in 2022 give more tools for monitoring entry-bans at the arcades.	Veikkaus controls access to its online casino and EMGs^3^ *via* digital ID identification. Veikkaus' casino has a customer register to which self-bans from the casino are recorded.
Germany	Land-based casinos and legal online gambling, including amusement machines with prizes (AWP) and sports betting since June 2021. Exceptions are lotteries with low event frequency and particular forms of horserace betting.	Casinos and organizers of sports betting and lotteries with a particular risk potential block persons who apply for self-exclusion. The organizers will immediately inform the gambler about the suspension in written form. In order to protect gamblers and to combat gambling addiction, the agents of public gambling are obliged to participate in the over-arching blocking system. For this purpose, the agents will immediately transmit the requests for self-blocking submitted to them to the organizer in the area in which the gambler is domiciled.	Central SE register (including providers of AWP since June 2021) (Spielsperrsystem OASIS)
Italy	Land-based casinos and online gambling	Self-exclusion from one online gambling website imposes exclusion from all legal online gambling providers. Exclusion from land-based casinos is requested and pertains to single casinos.	Central VSE register for online gambling only (since 2018)
Massachusetts (USA)	All three Massachusetts casino properties	The primary locations for VSE programs are the responsible gambling information centers formally branded as GameSense Info Centers. The Massachusetts Voluntary Self-Exclusion Program (MA-VSEP) provides interested patrons with three ways to self-exclude: (1) at the GameSense Info Center at each casino or with an MGC Gaming Agent at the casino when GameSense is closed, (2) at the Massachusetts Council on Gaming and Health [MACGH; formerly the Massachusetts Council on Compulsive Gambling (MCCG)] offices with a trained staff member, or (3) at the MGC main office in Boston with trained Gaming Commission staff^4^; To complete enrolment, interested individuals must present a government-issued photo ID, complete an enrolment application, and meet with an approved agent.	Central VSE register run by the MGC and called the Massachusetts Voluntary Self-Exclusion Program (MA-VSEP)
Norway	Norsk Tipping: mandatory offer of self-exclusion from various types of games, online games, games on terminals (EGMs), games on terminals in bingo halls, and other games^5^^,^ ^6^. Similar options for self-exclusion are provided by Norsk Rikstoto^7^. Self-exclusion may pertain to all games, a category of games or a single game.	All customers with the two monopolies can self-exclude with electronic ID. A substantial fraction of the gambling market in Norway is, however, unregulated. Providers operating from abroad provide on-line games adapted to Norwegian customers.	Norsk Tipping and Norsk Rikstoto control access *via* digital ID identification of all customers.
Sweden	Gambling online, in store and on the track as well as EGMs and land-based casinos	Self-exclusion from all gambling provided by gambling companies licensed in Sweden. Exclusion is confirmed with e-identification.	National self-exclusion register (www.spelpaus.se)
Victoria (Australia)	Co-regulatory schemes mainly cover land-based electronic gambling machines (EGM). As of 2022, online gambling providers run their own self-exclusion schemes, primarily through four websites (see https://gamblershelp.com.au/get-help/help-yourself/self-exclusion). An Australian National Self-Exclusion Register is being set up by the Australian Communications and Media Authority, which will require all internet gambling providers to exclude those listed on it from all internet gambling^8^.	Self-exclude from land-based gambling with an Easy Access Self-Exclusion (EASE) application. As part of the application, enrolees nominate all venues from which they want to be self-excluded; self-exclusion status requires the Melbourne casino's Responsible Gambling Team, the Community Clubs Victoria^9^, the Australian Hotel Association^10^, the Licensee of the Venue/s and/or the servants or agents of all those entities to take such action as is necessary to prevent the enrolee from entering the Restricted Gaming Areas and using gaming machines at the venues and to remove the enrolee from the Restricted Gaming Areas. The program must also ensure that there is the capacity to assist a self-excluder to also self-exclude from other gambling venues, and enrolees become ineligible for the period of self-exclusion to participate in any program for rewarding expenditure on gaming machines at the venue/s (“Loyalty Programs”)^11^.	No central VSE register for land-based gambling; for EGMs, there are three parallel self-exclusion systems in Victoria. A national self-exclusion register is being established for internet gambling.

1 Gambling locations are supermarkets, kiosks, gas stations etc. That mainly provide goods and services other than gambling but also sell lotteries and lottery games and operate EMGs in their foyers;

2 Veikkaus has two types of gambling arcades, Pelaamot and feel Vegas arcades. Both Offer EMGs, lotteries, lottery games, trotting games and sports betting. In Addition, feel Vegas gambling halls offer some table games such as Roulette; Veikkaus Oy (2020). Veikkaus annual and sustainability report 2020: https://cms.Veikkaus.fi/Site/Binaries/Content/Assets/Dokumentit/Vuosikertomus/2020/Annual_csr-Report_2020.pdf;

3 Before 2021, Veikkaus' EMGs required no compulsory authentication. In 2021, Veikkaus has gradually introduced compulsory authentication to its EMGs. The identification is done electronically. First, Veikaus introduced the compulsory identification at its agents (Kiosks, Supermarkets etc.) on 12.1.2021. Second, the compulsory identification was brought to the EMGs at Veikkaus' gambling arcades on 1.7.2021;

4 Massachusetts Gaming Commission, 2015;

5 https://www.Norsk-Tipping.no/Kundeservice/Spillevett/Spillepause-Utestenging/Hvordan-Kan-Jeg-Utestenge-Meg-Fra-Spill;

6 https://www.Norsk-Tipping.no/Spillevett/Verktoy;

7 https://www.Rikstoto.no/Nyttig-Informasjon/Regler-Og-Ansvar/Ansvarlig-Spill;

8 https://www.Acma.gov.au/National-Self-Exclusion-Register;

9 Community Clubs Victoria (*2021*, August 5). Venue Self-Exclusion Program: https://www.ccv.net.au/Venue-Self-Exclusion-Program/;

10 Australian Hotels Association Victoria (*2021*, August 5). Self-exclusion: https://www.Ahavic.com.au/Self-Exclusion/;

11 https://Gamblershelp.com.au/get-Help/Help-Yourself/Self-Exclusion/; https://www.Vcglr.vic.gov.au/Gambling/Gaming-Venue-Operator/Understand-Your-Gaming-license/Self-Exclusion-Program; for a ministerial direction concerning a self-exclusion program as a license condition, see for example, section 8 in: http://www.Gazette.vic.gov.au/Gazette/Gazettes2022/GG2022S195.pdf.

### Central register

Germany, Italy, Massachusetts, Norway, and Sweden maintain a central nation-/state-wide SE register, which enables an ID-based identification of self-excluded gamblers. These consumers ought to be denied access when trying to gamble at any venue or online service covered by the register. The Norwegian register covers all land-based gambling, the German and Swedish registers also include all licensed online services. The Italian national register covers self-exclusion from all online gambling providers holding a concession, while self-exclusion from land-based gambling is established separately for each casino; hence identification of self-excluded gamblers across sites is limited. This is also true for Finland, where customers can self-exclude from all online gambling *via* ID identification. If a customer, however, wants to self-exclude from the land-based gambling sites, (s)he needs to ask for an entry ban from the staff at each individual gambling site. In Massachusetts, the register applies to all three land-based casino properties. Victoria provides no central SE register; there are three separate systems operated on a co-regulatory basis by the casino, by the association of community and sports clubs, and by the hotels (taverns) association. For internet gambling, the Australian government is currently setting up a national SE register which will apply to all forms of online gambling.

### Length and termination

Options for the length of exclusion are manifold and vary across countries/states. In Finland, Italy, Norway and Sweden, customers can choose between various time periods and so-called “permanent” exclusion as it can be revoked after a certain period (Table 3). In Germany, SE lasts at least 1 year unless the enrolee applies for a shorter term, which must have a minimum length of 3 months. In Victoria, enrolment terms are 6, 12, 18 or 24 months, and in Massachusetts 12, 36 or 60 months. Temporal self-exclusion terminates automatically at the end of the set period in Finland, Italy, and Sweden. “Permanent” bans in Finland and Sweden are valid for a minimum of 1 year, and in Italy for 6 months; thereafter removal can be requested. In Finland, these bans will be lifted 3 months after the request for removal. In Massachusetts, enrolees must complete an “exit interview” from the SE program with a Massachusetts Gaming Commission-designated agent. In Italy and Germany, revocation of any SE requires a written application, and in Victoria, an enrolee must attend an interview with the relevant Industry body and produce written evidence that (s)he has received counseling from a qualified Problem Gambling Counselor.

**Table 3:** Summary of voluntary self-exclusion regulations (length, termination/revocation, utilization, control/enforcement by country/state.

**Country/State**	**Length**	**Termination/Revocation**	**Utilization**	**Control/Enforcement**
Finland	*Veikkaus land-based casinos and gambling arcades:* from 3 to 12 months. *Veikkaus online casino and other electronically transmitted games:* a temporary self-ban that can be set for a maximum of 1 year or an indefinite self-ban that is valid for a minimum of 1 year.	Veikkaus online casino: an indefinite ban is valid for the minimum of 1 year after which revocation is possible.	In 2020, 220 voluntary entry bans and restrictions at the Helsinki casino (426 in 2019, 486 in 2018, and 560 in 2017) ^1^.Voluntary entry bans to places “other than casino,” i.e., Veikkaus gambling venues (Pelaamot or Feel Vegas): 359 bans in 2020 (616 in 2019, 409 in 2018, and 359 in 2017)^1^. Veikkaus online casino: 23,650 effective game blocks on 31.12.2020. An increase of 2,700 effective blocks compared to 31.12.2019. The number of permanent blocks was 9,956 on 31.12. 2020 and 8,021 on 31.12.2019.	During 2021, the mandatory identification was introduced to EMGs at gambling locations and gambling arcades, allowing the player to practice an electronically controlled self-exclusion. Veikkaus has a goal to make all of its gambling identified by 2023.
Germany	At least 1 year unless the enrolee applies for a shorter term that may not be shorter than 3 months.	Not earlier than 1 year after self-exclusion and based on a written application. The venue from which the gambler has self-excluded decides on the applied revocation.	In 2020, 15% pathological gamblers with a lifetime diagnosis and 5% current pathological gamblers were registered in the SE register^2^.	Exclusion of customers is enforced by entrance control *via* personal ID and a request is made to the central register.
Italy	Temporary (30, 60, or 90 days) or permanent^3^. Exclusion from land-based casinos can be revoked after at least 3 months.	Automatically at the end of the set period; permanent exclusion can be revoked not earlier than 6 months after self-exclusion and must be specifically requested by email or to a call center.	Since February 2018, about 37,000 gamblers self-excluded from online gambling, of whom ~70% permanently (Osservatorio Gioco Online, School of Management del Politecnico di Milano, personal communication).	On the Service Charter for online gamblers is stated: “any non-compliance with a gambler's request to exclude himself causes the concessionaire to lose 10 points” (compared to 100 points that each concessionaire has at the start; the scoring system is used for the granting of concessions).
Massachusetts (USA)	Introductory enrolment terms are 12, 36 or 60 months.	After a patron's initial VSE period, if they wish to renew their MA-VSEP contract, they can select from the same 1-, 3-, or 5-year terms or select to be self-excluded for their lifetime. At any time after an individual's VSE period has expired, an enrolee can request that their name be removed from the VSE list. To finalize their removal from the list the individual must complete an “exit interview” with an MGC-designated agent (e.g., MACGH or MGC staff).	The Massachusetts Gaming Commission (MGC) reported that 1,020 individuals were enrolled in the Voluntary Self-Exclusion program as of 16 December 2021. Thirteen percent of enrolees formally un-enrolled when their term expired, and one third of those eventually re-enrolled in MA-VSEP.	People who violate their MA-VSEP contract are escorted from the gaming floor of the establishment when detected, and forfeit any money wagered, won, or lost, including money converted to wagering instruments. Forfeited monies do not return to the casino but are instead transferred to the MGC to be deposited into the Gaming Revenue Fund.
Norway	Options of permanent self-exclusion on various types of games and self-exclusion for a period of time (e.g., a week or a month)	“Permanent” self-exclusion can be revoked after a minimum of 12 months; shorter periods end automatically.	5,000 persons (unique gamblers) had self-excluded in 2019 (corresponding to 0.25% of all gamblers). An additional 24,000 had self-excluded for shorter periods. Among these, 11,000 had self-excluded for half a year, the remaining for a month 8,000 or a week 5,000^4^. According Norsk Rikstoto the number of gamblers that self-exclude temporarily for a period of a day, a week, a month or permanent ranged between 164 and 228 customers per month, corresponding to a maximum of 0.30% of all gamblers^5^.	Due to authentication with a digital ID, breaching of self-exclusion within the gambling monopoly is likely to be rare.
Sweden	Temporary for 1, 3 or 6 months or permanent	“Permanent” exclusion is valid for at least 12 months; shorter periods end automatically.	In August 2022, 79,700 people were registered on Spelpaus; of these 74% were men and 26% women; 68% excluded permanently (April 2020).	When a gambler logs in or registers with a licensee or when the licensee has to check recipients prior to individual marketing, a request is made as to whether a person is self-excluded or not. On average, 30.4 million inspections are performed per day^6^.
Victoria (Australia)	Self-exclude with EASE: 6, 12, 18 or 24 months	Self-exclude with EASE: the enrolee may revoke the period of this self-exclusion or reduce the period of self-exclusion but only: (1) after the expiration of a minimum period of 6 months from the commencement of the period of self-exclusion; (2) by arranging and attending another interview with the relevant industry body; (3) by producing written evidence that the person has received counseling from a qualified Problem Gaming Counselor in respect to revocation of self-exclusion; and (4) after signing and lodging with the Industry Bodies the Deed of Revocation of self-exclusion signed by the person.	Statistics on how many gamblers self-exclude from one or more gambling venues are not available. It is assumed that the numbers are not large.	Self-exclude with EASE: the Industry Bodies will contact the enrolees if they are detected breaching their self-exclusion; as part of the application enrolees need to accept that there is no legal responsibility, duty and/or obligation on the Industry Bodies, gaming operators, the Licensee of the Venue/s and/or their servants or agents to undertake any or all of the actions or things as authorized.

1 Veikkaus Oy (2020). Veikkaus annual and sustainability report 2020;

2 (25);

3 ADM (Prot. R.U. 44223/2018);

4 https://2019.Norsk-Tipping.no/Avbrekk-Tilpasset-Spillerne/;

5 E-mail communication with Bjørn Helge Hoffmann, Chief Advisor responsible gambling, Norks Tipping;

6 https://www.Spelinspektionen.se.

### Utilization

As gamblers can choose to engage in SE or not, its effectiveness in reducing harm mainly hinges on the individual gambler's motivation. The responsible gambling paradigm binds all gambling providers (governmental and licensed) to take responsible steps to prevent and minimize harm from gambling (28). Based on the assumption that problem gamblers are the ones that need to be protected from excessive gambling, SE could serve as an indicator of successful interventions (25). For Germany, the author estimated that 15 out of 100 individuals with a lifetime diagnosis of gambling disorder and 5 out of 100 individuals with a current gambling disorder self-excluded from gambling. Only Norway provides exact figures in relation to the total number of gamblers. In 2019, 29,000 persons had self-excluded from gambling, corresponding to 1.45% of the registered gamblers at Norsk Tipping. Including those who self-excluded at Norsk Rikstoto (0.3%) the proportion of gamblers who self-excluded amounted to 1.75%. For the other jurisdictions, only absolute numbers are reported, although no figures are reported for Victoria (Table 3).

### Enforcement

Self-exclusion can only be enforced routinely and completely if IDs of customers entering venues or logging on to gambling websites are checked against entries in a nation- or state-wide self-exclusion register. Such procedures have been implemented for land-based gambling in Massachusetts and Norway, for both online and land-based gambling in Sweden and most recently in Germany, and only for online gambling in Italy. Identification checks in Finland that were mandatory for Veikkaus online and land-based casinos have recently also been made mandatory when gambling at Veikkaus arcades and gambling locations. In Victoria, the industry self-regulatory office ought to theoretically contact the self-excluded gamblers if they are detected breaching their ban, but providers in parallel SE consortia are not liable for access checks. Indeed, enrolees must accept that there is no legal responsibility, duty and/or obligation on the industry bodies, gaming operators, the licensee of the venue/s and/or their servants or agents to undertake any actions that are authorized by the application as part of the application process. Customer penalties for violating SE are reported in Massachusetts. Enrolees violating their voluntary SE contract are escorted from the gaming floor of the establishment when detected and forfeit any money wagered, won, or lost, including money converted to wagering instruments. Forfeited monies are transferred to the Massachusetts Gaming Commission to be deposited into the Gaming Revenue Fund. In Sweden, the Gambling Authority has been reported to impose extensive fines on the gambling industry for violating the law regarding self-exclusion (Table 3).

## Discussion

### Self-exclusion and gambling policies

The seven jurisdictions included in this overview differ considerably in how gambling is regulated as well as in how SE is implemented and enforced. The reach and strength of the system, i.e., the extent to which there is actual enforcement of gambling providers' implementation of the system and the extent that they actually exclude, seem to vary with the polity's general policy balance between reducing gambling problems and increasing gambling revenue and building the economy. In Norway and Sweden, and to some extent also in Finland, with a strong focus on perceiving gambling as a public health and welfare issue, gambling—including the most addictive games—is strongly regulated through a state monopoly or licensing system where all gamblers are offered SE. With electronic ID for gambling, along with several other measures, SE appears to be better enforced than in Italy, Germany, Massachusetts, and Victoria.

Self-exclusion systems seem to be weak and thinly “patronized” in polities that have valued the revenue goal over the harm limitation goal. For instance, there have been considerable political scandals over gambling in Victoria, including money laundering and the laxness of state regulators, which resulted in a recent investigation into Crown Melbourne's business practices by a Victorian Royal Commission. In its report, the Commission concluded that “*Crown Melbourne has for many years consistently breached its Gambling Code and, therefore, a condition of its casino licence*” (35) (Volume 3, p. 37) because it failed to adequately interact with problem gamblers. Placing Victoria at one end and Norway and Sweden at the other end of the continuum of restrictive gambling policies appears to largely correspond with figures for gross gambling revenue per adult resident, with >900€ per head for Australia and < 400€ for Norway (2). Nevertheless, even in countries/states with strong gambling policies, a large part of the online gambling market is still not monopolized or licensed. The effectiveness of SE programs is limited by the possibility for customers to breach SE, for instance, by switching from licensed to unlicensed providers and often also by the lack of strict enforcement compelling the industry to follow SE regulations.

### Self-exclusion: A measure to reduce gambling harm in the population?

The gambling and SE regulations in all jurisdictions described above reveal several weaknesses that make SE ineffective in significantly reducing rates of gambling harm at the population level. First, in all jurisdictions, a substantial part of the gambling market is not monopolized or licensed. Self-excluded customers may continue to gamble at online providers that are not covered by the monopoly or that operate without a license in the particular jurisdiction. Second, with few exceptions there is a lack of consistent enforcement of the implementation of coherent SE regulations by both the state and the industry. Third, incoherent SE registers—if implemented at all—enable gamblers to circumvent the ban by, for instance, switching providers or changing from land-based to online formats, and vice versa; in fact, customers' breach of agreement seems to be the rule rather than the exception (10, 36). In a study by Nelson et al. (37), which surveyed gamblers under a lifetime exclusion agreement over an average period of 6.1 years, only 13% had not gambled at all since enrolment. A recent German study reported that 28.1% of gamblers were able to gamble on EGMs despite their SE (38). Presently, only Germany's, Norway's, and Sweden's SE systems and registers cover both land-based and licensed online gambling. The coverage of SE registers of the gambling market in the other countries/states is either low or a central register is not implemented at all. Fourth although data on this is not routinely available in any of the jurisdictions, the existing evidence points to a low rate of excluded problem gamblers. As problem gamblers are the target of SE measures, the effects on reducing gambling harm at the population level presumably remain low as long as the share of excluded problem gamblers among the total of problem gamblers is low. Fifth although studies investigating satisfaction with SE strategies reported generally positive ratings by the majority of respondents (39, 40), the decision to self-exclude or not is an individual choice. Hence, the effectiveness of SE in reducing harm at the population level mainly hinges on the individual gambler's motivation.

### Self-exclusion and the responsible gambling approach

Responsible gambling measures to prevent and minimize gambling harm, including but not limited to SE, have frequently been criticized as ineffective (41) and ethically problematic (3, 7). Rather than continuously extending gambling provisions, these authors propose limiting or eliminating certain forms of gambling-related harm. As measures which limit gambling conflict with the economic interests of both governmental and commercial gambling providers, their willingness to exercise strict regulation and enforcement is poor. This dilemma has been identified as the fundamental paradox in the gambling risk management agenda (42).

The option of gamblers banning themselves from gambling temporarily or permanently is inherently an “RG” measure in several senses of the term. First, it defines problematic gambling in terms of a dichotomy between a self-controlled course of action and behavior that is beyond the actor's self-control—why else would the gambler need to self-exclude? Second, it points to the gambler and the gambler's self-control as the aspect of the gambling transaction that is responsible for any problems—promotions and attractions from the gambling provider are out of the picture. Third, it offers an alibi to the provider and the gambling industry when harms occur—“look at what we are offering to avoid such situations.” Fourth, it is a measure that has proven to have only limited costs to the industry, in terms of how many gamblers take up the offer. This advantage is taken to a somewhat cynical length in Victoria, with the provision excluding possible payment of any damages by the gambling provider if the self-excluded person gambles anyway and loses again. Finally, SE is a rather peculiar strategy for limiting harm that has spread widely but is unique to gambling. There is no other attractive but problematic commodity or behavior to which an SE strategy has been applied as a major harm prevention strategy. In sum, by shifting the responsibility to the gambler, the state violates its responsibility to protect gamblers from gambling-related harm. This is particularly the case as about 60% of gambling revenue is estimated to come from problem gamblers (25, 43).

## Conclusion

In the included jurisdictions SE, as implemented, is a measure with only weak effects on public health. It is presumably the problem gamblers who need to be excluded, and although they are few, they account for a fairly large fraction of total gambling revenue. If a substantial proportion of the problem gamblers were excluded, it would have a significant impact on reducing gambling harm. In order to become an effective measure that protects those who are at risk for gambling problems and need to be prevented from financial, social, and psychological distress, SE utilization would need to be substantially increased by reforming legal regulations and exclusion conditions. This includes, among others, the closing of loopholes, i.e., minimizing the unlicensed part of the market; strict monitoring of providers' compliance with gambler protection regulations and early recognition activities by an independent body of control; a coherent SE register, including online and land-based gambling with ID checks of individuals at any time when initiating gambling. In addition, information for and motivation of gamblers, their relatives and gambling providers need to be intensified. Most importantly, the proportion of self-excluded problem gamblers among the total of problem gamblers needs to be established as a public health measure of effectiveness. An all-encompassing, well-functioning self-exclusion system could then be a part of a public health approach to effectively reducing gambling harm.

## Data availability statement

The original contributions presented in the study are included in the article/supplementary material, further inquiries can be directed to the corresponding author.

## Author contributions

LK, JKL, AMB, and LS designed the study. LK wrote the initial draft of the paper. RAV, SR, VEK, MH, IR, RR, TN, and JCÖ contributed by providing documents. All authors gave important feedback in revising the manuscript and approved the final version.

## Funding

This study was conducted within the framework of the Swedish program grant Responding to and Reducing Gambling Problems—Studies in Help-seeking, Measurement, Comorbidity and Policy Impacts (REGAPS) and the Bavarian Coordination Center for Gambling Issues [Landesstelle Glücksspielsucht Bayern (LSG)]. REGAPS received funding from the Swedish Research Council for Health, Working Life and Welfare (Forte; grant number 2016-07091). The LSG was funded by the Bavarian State Ministry of Public Health and Care Services. The State of Bavaria provides gambling services (lotteries, sports betting, and casino games) within the State gambling monopoly *via* the State Lottery Administration and provided funding for the Bavarian Coordination Center for Gambling Issues as an unrestricted grant. JCÖ, LK, SR, RAV, RR, and TN were supported by the Forte grant and LK, JKL, AMB, and LS by the Bavarian State Ministry of Public Health and Care Services. Funding for VEK and MH stems from a cooperation contract with the National Institute for Health and Welfare. The money is originally debited from the gambling monopoly retrospectively by the Finnish Ministry of Social Affairs and Health according to section 52 in the Lotteries Act; IR was supported by the Norwegian Institute of Public Health.

## Conflict of interest

The authors declare that the research was conducted in the absence of any commercial or financial relationships that could be construed as a potential conflict of interest.

## Publisher's note

All claims expressed in this article are solely those of the authors and do not necessarily represent those of their affiliated organizations, or those of the publisher, the editors and the reviewers. Any product that may be evaluated in this article, or claim that may be made by its manufacturer, is not guaranteed or endorsed by the publisher.
